# Clinical utility of *RAS* mutations in thyroid cancer: a blurred picture now emerging clearer

**DOI:** 10.1186/s12916-016-0559-9

**Published:** 2016-01-27

**Authors:** Mingzhao Xing

**Affiliations:** Laboratory for Cellular and Molecular Thyroid Research, Division of Endocrinology, Diabetes, and Metabolism, Department of Medicine, Johns Hopkins University School of Medicine, Baltimore, MD 21287 USA

**Keywords:** *RAS* mutation, Thyroid nodule, Thyroid cancer, Molecular marker, Diagnosis, Prognosis

## Abstract

*RAS* mutations play an important role in thyroid tumorigenesis. Considerable effort has been made in the last decade to apply *RAS* mutations as molecular markers to the clinical management of thyroid nodules and thyroid cancer. Yet, for the low diagnostic sensitivities and specificities of *RAS* mutations, when used alone, and for their uncertain role in the clinical outcomes of thyroid cancer, it has been unclear how to appropriately use them to assist the management of thyroid nodules and thyroid cancer. Studies from recent years, now added from the Alexander group, have shed light on this issue, making a blurred clinical picture now emerge clearer—*RAS* mutations, when combined with other genetic markers, have high diagnostic negative predictive values for thyroid cancer; cytologically benign thyroid nodules, including those positive for *RAS* mutations, have long-term clinical stability when non-surgically managed; and differentiated thyroid cancers harboring *RAS* mutations alone have an excellent prognosis. This progress in understanding *RAS* mutations in thyroid cancer is showing a major impact on molecular-based practice in the management of thyroid cancer.

Please see related research articles: http://dx.doi.org/10.1186/s12916-016-0554-1 and http://dx.doi.org/10.1186/s12916-015-0419-z

## Background

The *RAS* oncogene plays a fundamental role in human tumorigenesis [1]. Activating mutations in its three proto-oncogenes—*HRAS*, *KRAS* and *NRAS*—are found in nearly all human cancers. Thyroid cancer is one of the earliest cases where activating *RAS* mutations were discovered [2]. Today, the role of *RAS* oncogene in thyroid tumorigenesis has been well established [3–5]. Mutations in *RAS* genes occur, on average, in 30–45 % follicular thyroid cancer (FTC), 30–45 % follicular variant papillary thyroid cancer (FVPTC), 20–40 % poorly differentiated thyroid cancer (PDTC), 10–20 % anaplastic thyroid cancer, and rarely classical papillary thyroid cancer (PTC). *RAS* mutations also occur in 20–25 % benign follicular thyroid adenoma (FTA). Considerable interest has been drawn to the potential clinical utility of *RAS* mutations as diagnostic and prognostic molecular markers, among other molecular markers, for thyroid cancer [6]. Given these occurrence patterns in thyroid tumors, however, *RAS* mutations themselves have low diagnostic sensitivities and specificities. Also, the role of *RAS* mutations in the clinical behavior of thyroid tumor is uncertain. Consequently, the clinical picture of *RAS* mutations in thyroid cancer has been a blurred one.

### *RAS* mutations as diagnostic molecular markers for thyroid nodules

Clinical management of thyroid cancer usually starts with the evaluation of thyroid nodules, which is extremely common, with a prevalence of 50–70 % on ultrasonography [7, 8]. The diagnostic mainstay of thyroid nodules is fine needle aspiration biopsy (FNAB). In the last decade, much effort has been made to apply *RAS* mutations as diagnostic molecular markers to FNAB of thyroid nodules, particularly those with indeterminate cytology, which currently represents a major diagnostic challenge in clinical practice. Indeterminate thyroid cytology includes three categories—atypia of undetermined significance (AUS), follicular neoplasm (FN) and suspicious for malignancy [9]. Surgical treatment for thyroid nodules cytologically suspicious for malignancy is generally accepted because of its high preoperative malignancy probability (around 60–70 %) and the treatment decision seems to be relatively easy to make. How to treat AUS and FN, which have a moderate risk of malignancy (10–30 %), is a more challenging dilemma in treatment decision making. Resolution of this dilemma is believed to rely on the development of effective diagnostic molecular marker-based tests [6]. An ideal diagnostic molecular marker should have a 100 % or near-100 % sensitivity and specificity. Yet, *RAS* mutations have been proven to have inferior sensitivities and specificities for thyroid cancer. An effective strategy to apply *RAS* mutations to the diagnosis of thyroid cancer is their combinational use with other molecular markers, particularly genetic markers [6]. A prominent example is the panel of genetic makers, including *RAS* mutations, *BRAF* V600E mutation, *RET/PTC* and *PAX8/PPAR*γ rearrangements, which, in a single-center unblinded study, yielded increased diagnostic sensitivities for thyroid cancer in the indeterminate cytological setting to 88 % for AUS and 87 % for FN [10]. The sensitivity of this test on overall non-selected thyroid cytology was still high at 80 %, albeit lower, in a double-blinded multicenter study [11]. The recently discovered *TERT* promoter mutations [12] were shown to be diagnostically applicable to FNAB [13]. Expansion of the genetic diagnostic panel to include additional genetic markers, including *TERT* promoter mutations, has resulted in an improved sensitivity for the FN category to 90 %, bringing the negative predictive value to 96 % [14]. Indeterminate thyroid cytology consists mostly of follicular thyroid tumors and the malignant types are mainly FTC and FVPTC, which most commonly harbor *RAS* mutations. Therefore, *RAS* mutations are an important component that helps make the genetic panel diagnostically sensitive, propelling for high negative predictive values. It is expected that as the diagnostic efficiency of the genetic panel containing *RAS* mutations is confirmed and further improved, ideally through large blinded multicenter optimizing studies, it will become possible to use such a genetic panel to help reliably rule out malignancy for thyroid nodules.

### *RAS* mutations in cytologically benign thyroid nodules

Since FTA is a main thyroid tumor in the indeterminate cytology category and *RAS* mutations occur commonly in FTA, albeit less frequently than FTC and FVPTC, use of the diagnostic genetic panel is expected to result in a large number of *RAS* mutation-positive but cytologically benign or indeterminate thyroid nodules. How to appropriately manage these nodules has been unclear. Because of the preferential occurrence of *RAS* mutations in malignant over benign thyroid tumors and the established oncogenic functions of *RAS* mutations in human tumorigenesis, there is reasonable concern about the prognosis of cytologically benign or indeterminate thyroid nodules harboring *RAS* mutations. Consequently, some physicians may recommend surgical treatment for all *RAS* mutation-positive thyroid nodules. Others, on the other hand, may argue for non-surgical management and conservative follow-up for *RAS* mutation-positive but cytologically benign thyroid nodules, since non-surgical management of cytologically benign thyroid nodules is currently the standard management [15]. This confusion is a result of the lack of knowledge of the clinical behavior of such nodules in the long run.

The picture now becomes clearer with light shed by a marvelous recent study from Medici et al. of the Alexander group [16]. In this study, the authors investigated the diagnostic value of *RAS* mutations on FNAB for thyroid cancer and the clinical behavior of *RAS* mutation-positive thyroid tumors. As expected, the authors confirmed the low diagnostic sensitivity and specificity of *RAS* mutations when used alone. An important novel finding, however, is that a group of *RAS* mutation-positive but cytologically benign thyroid nodules that were available for non-surgical management and conservative monitoring all showed excellent stability without radiographic growth or adverse clinical consequences after a long-term clinical follow-up (mean 8.3 years). This provides the first evidence that *RAS* mutation-positive but cytologically benign thyroid nodules behave as true benign nodules and can be conservatively managed for long term. Given the expected rising number of such patients in the coming years, long-term non-surgical management of such nodules would likely have many benefits, such as cost savings, avoidance of potential surgical complications and preservation of the thyroid function. It should be noted, though, that the prognosis of *RAS*-positive but cytologically benign thyroid nodules in an even longer term beyond the follow-up time in the Medici et al. study remains unknown. Further studies with longer follow-up durations are needed to provide an even clearer picture. Also, the clinical significance of *RAS* mutations in AUS/FN nodules remains unclear.

### Benign clinical prognosis of benign thyroid nodules

Consistent with the limited role of *RAS* mutations in benign thyroid nodules discussed above, a recent publication in *BMC Medicine* by Medici et al., demonstrated the safety of long-term conservative clinical follow-up of cytologically benign thyroid nodules [17]. It has not been established how long benign thyroid nodules can be safely and conservatively followed clinically. Repeat evaluation of benign thyroid nodules at 1–2 years or even shorter intervals is currently common practice. This practice, however, has no direct evidence to support. In 1,254 patients with 1,819 cytologically benign thyroid nodules, with a median time of 1.4 years to first follow-up (ranging 0.5–14.1 years), the authors found no difference in malignancy or mortality between various follow-up intervals. Even though after three years of follow-up, some benign thyroid nodules could grow and even cause compressive symptoms requiring thyroidectomy in certain cases, the authors did not identify a difference in risk of malignancy. The results support the authors’ recommendation that the interval of repeat evaluation can be safely extended to three years, reducing the unnecessary visits and medical interventions. This will likely have a profound impact on the healthcare saving, given the large number of patients with benign thyroid nodules. These findings by Medici et al. are consistent with a recent Italian study similarly showing a generally long-term stability and benign prognosis of benign thyroid nodules [18]. These clinical findings also again support the notion that *RAS* mutation alone plays a limited role in the transformation of benign thyroid nodules as many of such nodules expectedly harbor *RAS* mutations [4].

### *RAS* mutations as prognostic molecular markers for thyroid cancer

Another important finding in the earlier Medici et al. study [16] is that, even when histologically confirmed to be malignant, the *RAS* mutation-positive tumors have limited aggressiveness; these are usually FVPTC without aggressive tumor behavior, such as extrathyroidal extension, lymph node metastasis and distant metastasis. These tumors are highly curable and have an excellent prognosis. This is in contrast to *BRAF* V600E mutation [19–21] or *TERT* promoter mutations [12, 13, 22, 23], which are associated with aggressiveness and poor clinical outcomes of thyroid cancer. Medici et al. confirmed the association of the *BRAF* V600E mutation with aggressive behavior of thyroid cancer in their study [16]. These results are consistent with the previous findings that, compared with the *BRAF* V600E mutation, *RAS* mutations were associated with better differentiation of thyroid cancer as reflected by normal or near-normal expression of thyroid iodide-handling genes in contrast to the considerable down-regulation of these genes with *BRAF* mutation [4, 24, 25]. Some studies showed an association between poor tumor behavior and *RAS* mutations in FTC [26] and PDTC [27, 28]. This likely reflects the coexistence of *RAS* mutations with additional oncogenic alterations, such as *TERT* promoter mutations; *RAS* mutations and *TERT* promoter mutations are significantly associated with each other in thyroid cancer [29] and coexistence of the two types of mutations was shown to be associated with a significantly higher tumor recurrence compared with *RAS* mutation alone in FTC [30], similar to the coexistence of *BRAF* V600E mutation with *TERT* promoter mutations that is associated with sharply increased aggressive clinicopathological behavior, tumor recurrence and patient mortality in PTC [23, 31]. *RAS* mutation alone is most likely associated with limited aggressiveness of thyroid cancer. This concept is in fact supported by a mouse model in which *Kras* mutation alone caused only benign thyroid neoplasia and its coexistence with *Pten* deletion in the PI3K pathway caused transformation of the tumor into FTC with metastasis [32].

One advantage of the expanded diagnostic genetic panel is that it tests not only *RAS* mutations, but also other important oncogenic genetic changes, including *TERT* promoter mutations. Therefore, coexistence of *RAS* mutations with additional oncogenic changes can be identified using such a genetic panel; the result should then be treated differently than finding *RAS* mutation alone in terms of prognostic significance. If only *RAS* mutations are found in differentiated thyroid cancer, it is reasonable and safe to assume a good prognosis.

## Conclusions

Studies in recent years together with the studies by Medici et al. [16, 17] have shed bright light on the role of *RAS* mutations in the clinical behavior of thyroid tumors and their value in assisting the management of thyroid nodules and thyroid cancer, making a previously blurred clinical picture now emerging clearer. Overall, cytologically benign thyroid nodules, even when harboring *RAS* mutations, have an excellent prognosis and can be safely and conservatively followed at relatively long time intervals; differentiated thyroid cancer, when harboring *RAS* mutations alone without other coexisting genetic alterations, generally lacks aggressive behaviors. Given this advancement in understanding *RAS* mutations, the following recommendations on their clinical use as diagnostic and prognostic molecular markers may be considered: 1) *RAS* mutations have an important diagnostic value as a component of a genetic marker panel that has high negative predictive values and can thus help diagnostically rule out thyroid malignancy; 2) *RAS* mutation-positive but cytologically benign thyroid nodules can be non-surgically managed and followed for long term; 3) *RAS* mutation-positive differentiated thyroid cancer has an excellent prognosis and can be treated with less aggressive measures, e.g. hemithyroidectomy, in the appropriate clinical settings; and 4) unless clinically indicated otherwise, *RAS* mutation-positive AUS or FN thyroid nodules may be treated with hemithyroidectomy and, if shown to be benign on additional molecular tests, such as gene expression classifier [33], may be considered to be non-surgically managed and conservatively followed. These uses of *RAS* mutations as molecular markers are applicable only when *RAS* mutation alone exists; if coexisting oncogenic genetic alterations are also found, such as *TERT* promoter mutations or mutations in the PI3K pathway, the tumor should be treated more aggressively in appropriate clinical settings.

## References

[1] Pylayeva-Gupta Y, Grabocka E, Bar-Sagi D (2011). RAS oncogenes: weaving a tumorigenic web. Nat Rev Cancer.

[2] Suárez HG, Du Villard JA, Caillou B, Schlumberger M, Tubiana M, Parmentier C (1988). Detection of activated ras oncogenes in human thyroid carcinomas. Oncogene.

[3] Xing M (2010). Genetic alterations in the phosphatidylinositol-3 kinase/Akt pathway in thyroid cancer. Thyroid.

[4] Xing M (2013). Molecular pathogenesis and mechanisms of thyroid cancer. Nat Rev Cancer.

[5] Howell GM, Hodak SP, Yip L (2013). RAS mutations in thyroid cancer. Oncologist.

[6] Xing M, Haugen BR, Schlumberger M (2013). Progress in molecular-based management of differentiated thyroid cancer. Lancet.

[7] Mazzaferri EL (1993). Management of a solitary thyroid nodule. N Engl J Med.

[8] Guth S, Theune U, Aberle J, Galach A, Bamberger CM (2009). Very high prevalence of thyroid nodules detected by high frequency (13 MHz) ultrasound examination. Eur J Clin Invest.

[9] Cibas ES, Ali SZ (2009). NCI Thyroid FNA State of the Science Conference. The Bethesda System For Reporting Thyroid Cytopathology. Am J Clin Pathol.

[10] Nikiforov YE, Ohori NP, Hodak SP, Carty SE, LeBeau SO, Ferris RL (2011). Impact of mutational testing on the diagnosis and management of patients with cytologically indeterminate thyroid nodules: a prospective analysis of 1,056 FNA samples. J Clin Endocrinol Metab.

[11] Beaudenon-Huibregtse S, Alexander EK, Guttler RB, Hershman JM, Babu V, Blevins TC (2014). Centralized molecular testing for oncogenic gene mutations complements the local cytopathologic diagnosis of thyroid nodules. Thyroid.

[12] Liu X, Bishop J, Shan Y, Pai S, Liu D, Murugan AK (2013). Highly prevalent TERT promoter mutations in aggressive thyroid cancers. Endocr Relat Cancer.

[13] Liu R, Xing M (2014). Diagnostic and prognostic TERT promoter mutations in thyroid fine-needle aspiration biopsy. Endocr Relat Cancer.

[14] Nikiforov YE, Carty SE, Chiosea SI, Coyne C, Duvvuri U, Ferris RL (2014). Highly accurate diagnosis of cancer in thyroid nodules with follicular neoplasm/suspicious for a follicular neoplasm cytology by ThyroSeq v2 next-generation sequencing assay. Cancer.

[15] Haugen BR, Alexander EK, Bible KC, Doherty GM, Mandel SJ, American Thyroid Association (ATA) Guidelines Taskforce on Thyroid Nodules and Differentiated Thyroid Cancer (2016). 2015 American Thyroid Association management guidelines for adult patients with thyroid nodules and differentiated thyroid cancer. Thyroid.

[16] Medici M, Kwong N, Angell TE, Marqusee E, Kim MI, Frates MC (2015). The variable phenotype and low-risk nature of RAS-positive thyroid nodules. BMC Med.

[17] Medici M, Liu X, Kwong N, Angell TE, Marqusee E, Kim MI, et al. Long-interval versus short-interval follow-up of cytologically benign thyroid nodules: a prospective cohort study. BMC Medicine. 2016; doi:10.1186/s12916-016-0554-1 10.1186/s12916-016-0554-1 PMC4730757 26817603

[18] Durante C, Costante G, Lucisano G, Bruno R, Meringolo D, Paciaroni A (2015). The natural history of benign thyroid nodules. JAMA.

[19] Xing M, Westra WH, Tufano RP, Cohen Y, Rosenbaum E, Rhoden KJ (2005). BRAF mutation predicts a poorer clinical prognosis for papillary thyroid cancer. J Clin Endocrinol Metab.

[20] Xing M, Alzahrani AS, Carson KA, Viola D, Elisei R, Bendlova B (2013). Association between BRAF V600E mutation and mortality in patients with papillary thyroid cancer. JAMA.

[21] Xing M, Alzahrani AS, Carson KA, Shong YK, Kim TY, Viola D (2015). Association between BRAF V600E mutation and recurrence of papillary thyroid cancer. J Clin Oncol.

[22] Vinagre J, Almeida A, Pópulo H, Batista R, Lyra J, Pinto V (2013). Frequency of TERT promoter mutations in human cancers. Nat Commun.

[23] Xing M, Liu R, Liu X, Murugan AK, Zhu G, Zeiger MA (2014). BRAF V600E and TERT promoter mutations cooperatively identify the most aggressive papillary thyroid cancer with highest recurrence. J Clin Oncol.

[24] Giordano TJ, Kuick R, Thomas DG, Misek DE, Vinco M, Sanders D (2005). Molecular classification of papillary thyroid carcinoma: distinct BRAF, RAS, and RET/PTC mutation-specific gene expression profiles discovered by DNA microarray analysis. Oncogene.

[25] Cancer Genome Atlas Research Network (2014). Integrated genomic characterization of papillary thyroid carcinoma. Cell.

[26] Fukahori M, Yoshida A, Hayashi H, Yoshihara M, Matsukuma S, Sakuma Y (2012). The associations between RAS mutations and clinical characteristics in follicular thyroid tumors: new insights from a single center and a large patient cohort. Thyroid.

[27] Garcia-Rostan G, Zhao H, Camp RL, Pollan M, Herrero A, Pardo J (2003). RAS mutations are associated with aggressive tumor phenotypes and poor prognosis in thyroid cancer. J Clin Oncol.

[28] Volante M, Rapa I, Gandhi M, Bussolati G, Giachino D, Papotti M (2009). RAS mutations are the predominant molecular alteration in poorly differentiated thyroid carcinomas and bear prognostic impact. J Clin Endocrinol Metab.

[29] Liu R, Xing M. *TERT* promoter mutations in thyroid cancer. Endocr Relat Cancer. 2016 Jan 5. pii: ERC-15-0533. [Epub ahead of print] 10.1530/ERC-15-0533 PMC4750651 26733501

[30] Muzza M, Colombo C, Rossi S, Tosi D, Cirello V, Perrino M (2015). Telomerase in differentiated thyroid cancer: promoter mutations, expression and localization. Mol Cell Endocrinol.

[31] Xing M, Liu R, Bishop J (2014). TERT promoter and BRAF mutations cooperatively promote papillary thyroid cancer-related mortality. Thyroid.

[32] Miller KA, Yeager N, Baker K, Liao XH, Refetoff S, Di Cristofano A (2009). Oncogenic Kras requires simultaneous PI3K signaling to induce ERK activation and transform thyroid epithelial cells in vivo. Cancer Res.

[33] Alexander EK, Kennedy GC, Baloch ZW, Cibas ES, Chudova D, Diggans J (2012). Preoperative diagnosis of benign thyroid nodules with indeterminate cytology. N Engl J Med.

